# Assessing the risk and disease burden of *Clostridium difficile* infection among patients with hospital-acquired pneumonia at a University Hospital in Central China

**DOI:** 10.1007/s15010-017-1024-1

**Published:** 2017-05-11

**Authors:** Chunhui Li, Juping Duan, Sidi Liu, Xiujuan Meng, Chenchao Fu, Cui Zeng, Anhua Wu

**Affiliations:** 1 Infection Control Center, Xiangya Hospital, Central South University, Changsha, 410008 Hunan China; 2 Department of Pharmacy, Changsha Hospital of Traditional Chinese Medicine, Changsha, 410000 China

**Keywords:** *Clostridium difficile* infection (CDI), Disease burden, Hospital-acquired pneumonia, Broad-spectrum antibiotics

## Abstract

**Purpose:**

Hospital-acquired pneumonia (HAP) remains one of the major hospital-acquired infections in China. Antibiotic treatment of HAP may lead to subsequent *Clostridium difficile* infection (CDI). Baseline data on the occurrence of CDI among HAP patients in China are currently unavailable. This study examines the risk and disease burden of CDI among HAP hospitalized patients (HAP-CDI).

**Methods:**

We conducted a prospective study among ICU patients with HAP and hospital-onset diarrhea from January 2014 to December 2014 in a teaching hospital in China. All stool specimens were cultured for *C. difficile* which were typed by MLST. We used univariate and multivariable regression analyses to identify risk factors of HAP-CDI.

**Findings:**

In total, 369 patients who met the inclusion criteria were enrolled. Thirty-two patients tested *C. difficile* positive. Among the isolated *C. difficile* strains, 90.63% (29/32) isolates were toxinogenic. Various MLST types were identified. The incidence of HAP-CDI was 11.67/10,000 patient days (95% CI, 7.97–16.55). Nineteen patients died from complications. The attributable mortality rate was 5.15% (19/369). The mortality rate of HAP-CDI group was 13.79% which was higher than HAP-non-CDI group. Univariate analyses demonstrated that old age, receiving antibiotics (OR = 8.70) and glucocorticoids (OR = 7.71) 1 month prior to hospitalization, respiratory failure (OR = 3.28) and receiving antimicrobials during hospitalization (OR = 1.15) were the risk factors associated with CDI. Multivariate conditional logistic regression analysis demonstrated the similar results.

**Conclusion:**

CDI was common among patients discharged from hospital for HAP at a university hospital. Prevention of the spreading of *C. difficile* among hospitalized patients is urgently needed.

**Electronic supplementary material:**

The online version of this article (doi:10.1007/s15010-017-1024-1) contains supplementary material, which is available to authorized users.

## Introduction

*Clostridium difficile* infection (CDI) is a leading cause of hospital-acquired infection (HAI) worldwide [1]. *Clostridium difficile* is one of the pathogens monitored for HAI studies in the United States [2]. *Clostridium difficile* has surpassed multi-drug resistant organisms (MDROs) such as methicillin-resistant staphylococcus aureus (MRSA) as the most common pathogen causing HAI. CDI causes more than 450,000 cases and 29,000 deaths in the United States each year [2–4]. Approximately 172,000 CDI cases occur each year across the 27 countries of the European Union (EU) [5].

Hospital-acquired pneumonia (HAP) is the most prevalent HAI in China [6]. HAP is a major cause of infection-associated morbidity and mortality in many countries including China. HAP leads to increased antibiotic treatment in hospitals [7]. MDROs were frequently isolated from patients with HAP in China [8, 9]. Broad-spectrum antibiotics are often empirically prescribed to treat HAP.

Broad-spectrum antibiotics, chemotherapeutics and proton pump inhibitors (PPI) induce dysbiosis of intestinal flora, which leads to CDI [10]. Treatment with antibiotics such as third-generation cephalosporins, broad-spectrum penicillins and fluoroquinolones is risk factor for CDI [11]. The treatment with moxifloxacin and gatifloxacin was linked to the outbreaks of CDI caused by hypervirulent strains [12].

The increase of the prescription of broad-spectrum antibiotic is linked to the increased number of hospitalizations of patients with HAP. The incidence of antimicrobial-associated complications [e.g., antimicrobial-associated diarrhea (AAD)] is also rising [13]. CDI may lead to severe outcome among hospitalized patients, especially HAP patients in China. However, few reports have described the epidemiology of HAP complicated by CDI. The prevalence of hospital-onset CDI and the incidence rate among HAP patients in China are currently unavailable. Epidemiologic study of CDI among HAP patients is needed for infection prevention. This study aimed to assess risk factors, mortality and incidence rate resulting from CDI among hospitalized patients treated for HAP.

## Materials and methods

### Study design, population, inclusion and exclusion criteria

We conducted a prospective study on patients with HAP presenting subsequent hospital-onset diarrhea (HOD) from January 2014 to December 2014 in four ICUs. All study subjects were adult patients admitted to Xiangya Hospital, a 3500-bed tertiary university hospital with approximately 100,000 admissions annually in China. Patients included were patients diagnosed with HAP which is defined as parenchymal lung infection that occurs ≥48 h after admission. Patients who were incubated at the time of admission were excluded. HAP which was defined in 2010 [14] includes ventilator-associated pneumonia (VAP). The definition of HOD is diarrhea occurring ≥48 h after hospital admission [15]. Patients suffering from ≥3 HOD episodes within 24 h were suspected for CDI and were enrolled in the study [16]. CDI was diagnosed by the presence of toxigenic *C. difficile* in the stool. Patients were divided into HAP-CDI group (presence of toxigenic *C. difficile* in the stool) and HAP-non-CDI group (absence of *C. difficile* in the stool). Data were extracted from the patient’s medical records using a structured report form. Variables analysis included demographic variables, underlying conditions, pathogen of HAP (only bacteria), choice and duration of anti-HAP therapy and clinical outcomes. We abstracted record information from the patient’s hospital number and assigned each patient with a unique study ID regardless of CDI test results in an Epi Data database.

### *Clostridium difficile* test and CDI case definition

Stool samples were collected from suspected CDI patients and tested for *C. difficile*. First, samples were transferred onto CDMN agar (OXOID) in anaerobic airtight containers (OXOID). Identification of isolates was based on odor and the appearance of colonies. The final confirmation of *C. difficile* was made by commercially available latex agglutination test [glutamate dehydrogenase (GDH)] (OXOID) and PRO DISK (Remel). The toxin genes *tcdA*, *tcdB*, *cdtA* and *cdtB* were detected by PCR according to prior recommendations [17–19]. Patients whose stool samples tested positive for toxin-producing *C. difficile* by culture and PCR were diagnosed with CDI. We excluded patients with diarrhea onset occurring less than 48 h following hospital admission.

### Multilocus sequence typing (MLST)

MLST was performed and analyzed for the toxigenic and non-toxigenic *C. difficile* strains according to the previous publications. Briefly, MLST with seven housekeeping genes *adk*, *atpA*, *dxr*, *glyA*, *recA*, *sodA* and *tpi* were performed on all isolates as described previously by Griffiths et al. [20].

#### Data analysis

Epidemiologic, clinical and laboratory data were linked by study ID, verified and analyzed using SPSS 20.0 (IBM). The incidence rate of CDI was calculated as CDI cases/10,000 patient days [15]. Descriptive statistics and univariate analyses were performed. Continuous variables were expressed by mean ± SD and were compared using *t* test; categorical variables were expressed as proportions and were compared using the *χ* ^2^ test and Fisher’s exact test when necessary to assess differences between patient populations. Multivariate logistic regression analysis was used to assess parameters associated with acquisition of *C. difficile.* Two-tailed *P* value of less than 0.05 was considered significant. We performed conditional logistic regression, eliminating variables through a step-wise approach if the *P* value for an independent variable was >0.1 Odds ratios and 95% confidence intervals were calculated.

## Results

**Fig. 1:**
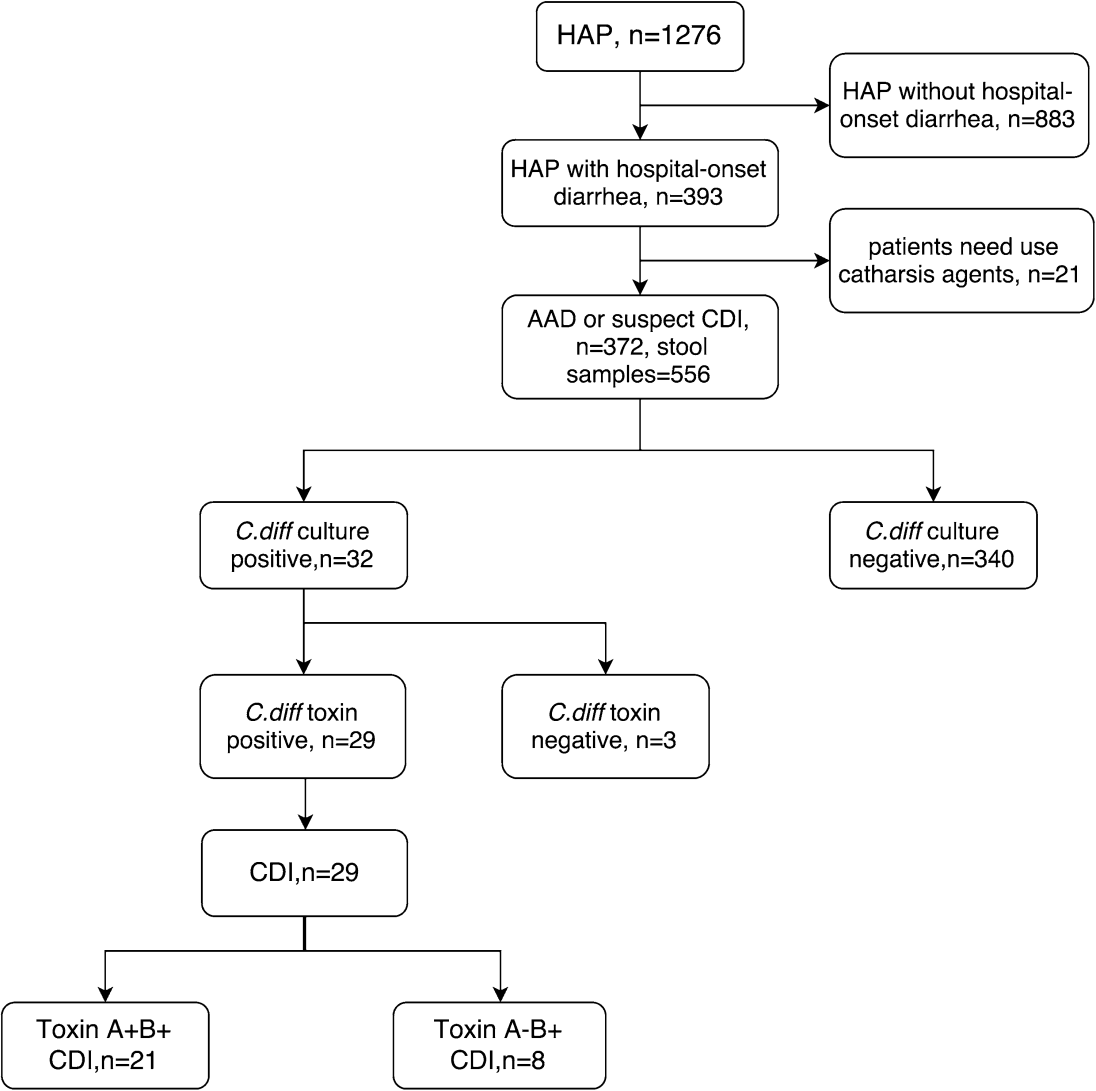
Enrollment of 29 HAP with *C. difficile* infection (CDI) patients and 340 HAP without CDI patients during 12 months of surveillance (from January 2014 to December 2014) in Xiangya Hospital, China

Three hundred and sixty-nine patients were enrolled in the study. Three hundred and seventy-two patients met the definition of HAP with suspect CDI. Twenty-nine patients were diagnosed with CDI (HAP-CDI group). Two patients were positive for *C. difficile*, but negative for toxin. Three hundred and forty patients were identified as HOD without *C. difficile* (HAP-non-CDI group) (Fig. 1). The mean age of all enrolled patients was 58.07 years, and 72.63% (268/369) were male. The average length of hospital stay was 20.70 days (range 4–77 days). The incidence rate of HAP with CDI was 11.67/10,000 patient days (95% CI, 7.97–16.55). Nineteen patients died from complications. And the attributable mortality rate of the enrolled group was 5.15% (19/369). The mortality rate of the HAP-CDI group was 13.79% (4/29), which was higher than HAP-non-CDI group (4.41%, 15/340) (*P* value <0.05). However, we could not conclude that CDI solely contributed to increased mortality.

### Risk factors for HAP-CDI

Univariate analyses were conducted to compare the differences of the HAP-CDI group and HAP-non-CDI group on demographic, clinical and baseline characteristics of patients (Table 1). Patients with HAP-CDI (mean age 64.83, range 27–90 years) were older than HAP-non-CDI patients (mean age 57.49, range 18–94 years) (*P* value <0.05). About 60% of the patients in the HAP-CDI group were over 65. The HAP-CDI group required longer periods of hospitalization (mean 28 versus 20 days) (*P* value <0.001), and suffered from more underlying conditions causing respiratory failure (34.48 versus 13.82%, OR = 3.28) (*P* value <0.05). Higher percentage of the patients in HAP-CDI group received antimicrobials (79.31 versus 30.59%, OR = 8.70) and glucocorticoids (27.59 versus 4.71%, OR = 7.71) 1 month prior to hospitalization compared to HAP-non-CDI group (all *P* values <0.01). More patients in the HAP-CDI group received antimicrobials during hospitalization (100 versus 87.35%, OR = 1.15, *P* value <0.05). Multivariate conditional logistic regression analysis demonstrated that older age, treatment with antibiotics and glucocorticoids 1 month prior to hospitalization and total parenteral nutrition (TPN) were risk factors associated with the development of CDI among HAI cases.

**Table 1:** Univariate and multivariate analyses regarding the demographic, risk factors and mortality rate of HAP-CDI and HAP-non-CDI groups

Characteristics	HAP-CDI (*n* = 29)		HAP-non-CDI (*n* = 340)		Univariate		Multivariable	
	*n*	%	*n*	%	OR (95% CI)	*P* value	OR (95% CI)	*P* value
Male	24	82.76	244	71.76	1.889 (0.700–5.093)	0.202		
Age mean ± SD	64.83 ± 14.83		57.49 ± 15.45			0.014	1.054 (1.014–1.096)	0.008
Age group								
18–40	2	6.90	41	12.06	0.540 (0.124–2.356)	0.406		
41–64	10	34.48	185	54.41	0.441 (0.199–0.976)	0.039		
>65	17	58.62	108	31.76	3.043 (1.404–6.595)	0.003		
Hospital duration (days) (mean ± SD)	28.55 ± 16.38		20.03 ± 8.48			<0.001	1.050 (1.002–1.100)	0.041
Underlying conditions								
Diabetes mellitus	5	17.24	52	15.29	1.154 (0.421–3.161)	0.781		
Malignancy	3	10.34	20	5.88	1.846 (0.515–6.624)	0.34		
Hematopathy	1	3.45	4	1.18	3.000 (0.324–27.759)	0.31		
Respiratory failure	10	34.48	47	13.82	3.281 (1.437–7.490)	0.003		
Renal insufficiency	3	10.34	26	7.647	1.393 (0.395–4.914)	0.604		
Cardiac insufficiency	1	3.45	48	14.12	0.217 (0.029–1.634)	0.104		
1 month prior hospital admission								
Used antibiotics	23	79.31	104	30.59	8.699(3.44–21.995)	<0.001	7.298 (2.284–23.319)	0.001
Used immunosuppressant	0	0	7	2.06	1.021 (1.005–1.037)	0.435		
Used glucocorticoids	8	27.59	16	4.71	7.714 (2.964–20.079)	˂0.001	6.331 (1.390–28.841)	0.017
Treatments and procedures during hospital stay								
Surgery	9	31.03	111	32.65	0.928 (0.409–2.105)	0.859		
Tube feeding	20	68.97	185	54.41	1.862 (0.824–4.207)	0.13		
TPN	18	62.07	162	47.65	1.798 (0.824–3.921)	0.136	3.944 (1.306–11.912)	0.015
Enema	0	0	5	1.47	1.015 (1.002–1.028)	0.511		
Enteroscopy	2	6.90	23	6.76	1.021 (0.228–4.564)			
Antibiotics use	29	100	297	87.35	1.145 (1.099–1.192)	0.042		
PPI use	27	93.10	299	87.94	1.851 (0.424–8.075)	0.406		
Mortality rate	4	13.79	15	4.55	3.467 (1.070–11.232)	0.028		

*TPN* total parenteral nutrition, *PPI* proton pump inhibitor, *Surgery* had surgery during hospitalization

### Antimicrobials use in HAP

**Fig. 2:**
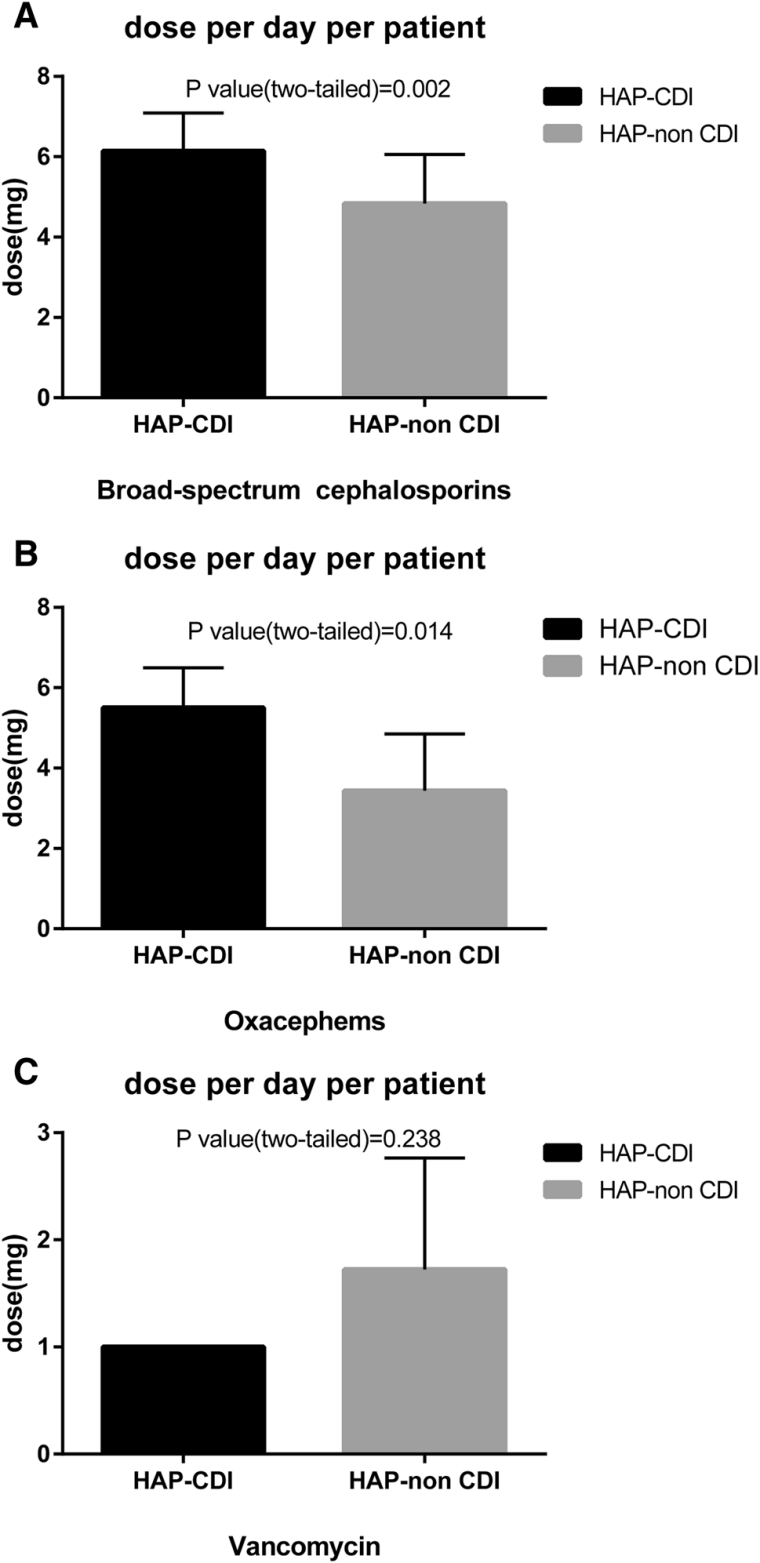
Compared dose per day per patient for extended-spectrum cephalosporins (**a**), oxacephems (**b**) and vancomycin (**c**) using among HAP-CDI and HAP-non-CDI group patients

88.35% HAP and 100% the HAP-CDI patients received one or more antimicrobial therapy (Table 2). The top three antimicrobials taken by HAP patients during hospitalization were extended-spectrum cephalosporins + inhibitors (40.11%), carbapenem (39.30%) and antipseudomonal penicillins + inhibitors (34.42%) (all the antimicrobial categories and agents of this study are shown in supplemental Table 1). The top three bacteria causing HAP were *Acinetobacter baumannii* (37.67%), *Klebsiella pneumoniae* (14.36%), *Pseudomonas aeruginosa* (10.30%) (the proportion of bacteria causing HAP among two groups are shown in Supplemental Fig. 1). The univariate analysis results showed that patients who received extended-spectrum cephalosporins and oxacephems were at higher risk for developing CDI (*P* value <0.05). Receiving intravenous vancomycin did not show protective effect against developing CDI (*P* value >0.05). Compared to the HAP-non-CDI group, the HAP-CDI group received longer period and higher dosage of extended-spectrum cephalosporin treatment (*P* value <0.05) and more doses per day per patient for extended-spectrum cephalosporins and oxacephems (*P* value <0.05). The dose of vancomycin use did not present significant difference between the two groups (*P* value >0.05) (Fig. 2).

**Table 2:** Crude odds ratios and 95% confidence analyses regarding therapeutic antimicrobials and bacterial of HAP for HAP-CDI and HAP-non-CDI groups

Characteristics	HAP-CDI (*n* = 29)		HAP-non-CDI (*n* = 340)		OR (95% CI)	*P* value
	No./days/dose (mg)	%	No./days/dose (mg)	%		
Therapeutic antimicrobials for HAP						
Broad-spectrum cephalosporins	14	48.28	91	26.76	2.55 (1.186–5.499)	0.014
Duration of use days per patient	7.29 + 3.41		5.14 + 3.75			0.04
Total dose per patient	44.14 + 23.30		24.73 + 19.41			0
Broad-spectrum cephalosporins + inhibitors	13	44.83	135	39.715	1.234 (0.575–2.647)	0.589
Duration of use days per patient	8.89 + 6.15		7.07 + 4.95			0.463
Total dose per patient	85.83 + 75.04		62.16 + 47.54			0.132
Carbapenem	13	44.83	132	38.82	1.28 (0.597–2.748)	0.525
Duration of use days per patient	6.38 + 3.48		5.36 + 3.66			0.95
Total dose per patient	16.31 + 9.33		13.56 + 11.43			0.643
Antipseudomonal penicillins + inhibitors	10	34.48	117	34.41	1.003 (0.452–2.228)	0.994
Duration of use days per patient	7.40 + 4.88		6.61 + 5.08			0.933
Total dose per patient	93.15 + 69.55		85.96 + 68.01			0.649
Vancomycin	3	10.34	51	15.00	0.654 (0.191–2.241)	0.496
Duration of use days per patient	5.33 + 2.89		5.81 + 3.58			0.726
Total dose per patient	5.6 + 2.43		7.46 + 7.02			0.315
Fluoroquinolones	5	17.24	75	22.06	0.736 (0.272–1.995)	0.546
Duration of use days per patient	4.4 + 2.88		5.64 + 4.14			0.465
Total dose per patient	1.92 + 1.29		2.48 + 2.26			0.226
Oxacephems	4	16.00	16	4.71	3.240 (1.007–10.426)	0.038
Duration of use days per patient	5.5 + 0.58		4.19 + 3.04			0.41
Total dose per patient	27.5 + 7.00		14.25 + 11.00			0.036
Non-broad cephalosporins	1	3.45	15	4.412	0.774 (0.099–6.076)	0.807
Duration of use days per patient	1.00		4.6 + 2.87			–
Total dose per patient	4.00		21.67 + 16.46			–
Aminoglycosides	0	0	9	2.65	1.027 (1.009–1.045)	0.375
Duration of use days per patient	0		7.33 + 4.06			–
Total dose per patient	0		4.46 + 3.41			–
Metronidazole	0	0	1	0.29	1.003 (0.997–1.009)	0.77
Duration of use days per patient	0		1			–
Total dose per patient	0		0.4			–
Pathogen of HAP						
*Acinetobacter baumannii*	15	51.72	124	36.47	1.866 (0.872–3.995)	0.104
*Klebsiella pneumoniae*	7	24.14	46	13.53	2.034 (0.822–5.030)	0.118
*Pseudomonas aeruginosa*	2	6.90	36	10.59	0.626 (0.143–2.740)	0.53
*E. coli*	4	13.79	18	5.29	2.862 (0.900–9.106)	0.064
*Staphylococcus aureus*	1	3.45	13	3.82	0.898 (0.113–7.121)	0.919

### Molecular characteristics of *C. difficile*

Thirty-two isolates of *C. difficile* were isolated from 556 stool samples, among which 29 were toxigenic. Among toxigenic *C. difficile* isolates, 21 (72.41%) were toxin A+B+ strains, 8 were toxin A−B+ strains. Nine different STs were observed by analyzing all isolates including toxigenic and non-toxigenic *C. difficile* strains by MLST. ST54 (*n* = 8, 20%) was the most common MLST type, followed by ST37 (*n* = 5), ST3 (*n* = 3) (Supplemental Fig. 2). Neither ST-1/RT027 nor ST-11/RT 078 was detected during the study period. The binary toxin genes *cdtA* and *cdtB* were not found in any toxigenic or non-toxigenic strains.

## Discussion

HAP is one of the leading HAIs worldwide and is associated with an elevated morbidity, mortality and increased hospital costs. HAP patients are also at risk for acquiring MDROs given that antimicrobial therapy especially combination therapy with two or more antimicrobials may be prescribed to treat pathogens potentially resistant to single antibiotic. However, antimicrobials are also associated with increased incidence of CDI with prolonged therapy [21].

Few studies have been conducted on the epidemiology of CDI in HAP population. Our study demonstrated that the incidence rate of HAP-CDI was 11.67 per 10,000 patients. Limited reports showed that CDI prevalence in HAP patients was 10.8 CDI cases per 1000 pneumonia discharges. CDI in HAP patients was associated with a significant increase in mortality, length of hospital stay, and treatment cost [22]. Our study also revealed longer hospital stay among HAP-CDI group compared to HAP-non-CDI group. The finding of increased hospital stay in the HAP-CDI group is consistent with a study by Gabriel et al [23]. In our study, ICU patients with HAP and subsequent CDI had a greater probability of deaths compared to HAP patients who did not develop CDI. The contribution to morbidity rate by CDI was not estimated in this study due to the lack of clinical data. However, previous studies reported that CDI had a significant negative impact on patient survival [24].

CDI-associated hospitalizations are longer, more costly, and have morbidity rate compared with hospitalized patients without CDI in middle-aged and senior population [25]. Previous studies demonstrated that risk factors for CDI included old age, prior healthcare exposures, underlying conditions including chronic disease and antimicrobial exposures [26]. This study confirmed that age over 65 years, respiratory failure, antimicrobial and glucocorticoid exposures as well as TPN were risk factors for CDI. In particular, the most significant risk factor for CDI was exposure to antimicrobials before hospitalization, with a high odds ratio over 7 between HAP-CDI and HAP-non-CDI groups.

Broad-spectrum antibiotic use caused decreases in indigenous bacterial diversity and plays an important role in the pathogenesis of CDI. Nearly all classes of antimicrobials have been associated with CDI. Clindamycin, third-generation cephalosporins, fluoroquinolones usage were considered to pose the highest risk for CDI [1]. Brown and colleagues reported that the risk for CDI was more than tripled after any antimicrobial exposure [27]. Antimicrobials that cause minimal disruption of the anaerobic microflora (e.g., aztreonam) did not promote CDI in mice or hamsters [28]. The risk caused by antimicrobial exposure is dose-dependent and increases with prolonged antimicrobial use and with combination therapy [29]. Our study only revealed that broad-spectrum cephalosporins and oxacephems were associated with increased risk of CDI among HAP patients. Different from previous studies, carbapenem, broad-spectrum cephalosporins + inhibitors and fluoroquinolones did not present increased risk for CDI [26]. Prolonged treatment and higher dosage of cephalosporins and oxacephems increased the risk of developing CDI. Antimicrobials that are active against *C. difficile* decrease the risk of colonization and infection during their use (e.g., oral vancomycin) [30]. However, intravenous vancomycin is ineffective against *C. difficile* [31]. In our study, intravenous vancomycin use did not protect patients from developing CDI.

Most of the toxigenic *C. difficile* isolates identified in this study were toxin A+B+. ST54 was the most common epidemic strain in China [32]. Neither hypervirulent *C. difficile* ST-1/RT027 nor ST-11/RT 078 was detected during the study period. Binary toxin genes *cdtA* and *cdtB* were not detected in any toxigenic and non-toxigenic strains isolated in this study.

This study presents several limitations. First, this study was conducted in a single medical center. The sample size of the HAP-CDI group was small. Study on larger sample size may produce more comprehensive results. Moreover, the financial burden associated with CDI was not calculated. Third, patient-to-patient transmission of CDI was not assessed. No information was collected to indicate whether or not a *C. difficile* outbreak was present at the time of the study.

Multi-faceted programs of CDI prevention and control were effective and cost-saving. Recently, a review on guidelines, strategies, and recommendations on infection prevention and control of *C. difficile* was published [33]. CDI infection prevention and control are critical to cost saving, quality improvement in healthcare and patient safety. Awareness of CDI prevention and control has increased in countries with high incidence rate of CDI such as the United States, Canada, Europe and the Western Pacific [33]. However, the epidemiology of *C. difficile* has not been extensively examined in China and awareness of CDI is lacking. Information of epidemiology of CDI in China is critical for the disease prevention and control.

## Conclusions

In conclusion, this study described the molecular epidemiology of HAP-CDI. Risk factors for the development of CDI among HAP patients were identified. The usage of antimicrobials played an important role in the pathogenesis of CDI. Antimicrobials should be prescribed with caution for HAP patients in hospitals. Findings of this study will help establish Antimicrobial Stewardship Program (ASP) and develop HAI surveillance, prevention and control programs and guidelines on CDI in China.

## Electronic supplementary material

Below is the link to the electronic supplementary material.

Supplemental Fig. 1. The proportion of bacterial for HAP among HAP-CDI group (A) and HAP-nonCDI group (B) (TIFF 10795 kb)

Supplemental Fig. 2. Number of different MLST types and positive toxin gene on *C. difficile* (TIFF 8133 kb)

Supplementary material 3 (DOCX 13 kb)
