# Supplementary figures and images for: Assessing the risk and disease burden of Clostridium difficile infection among patients with hospital-acquired pneumonia at a University Hospital in Central China
Source: Infection. 2017 May 11;45(5):621–8. doi: 10.1007/s15010-017-1024-1 (PMC5630651; doi:10.1007/s15010-017-1024-1)

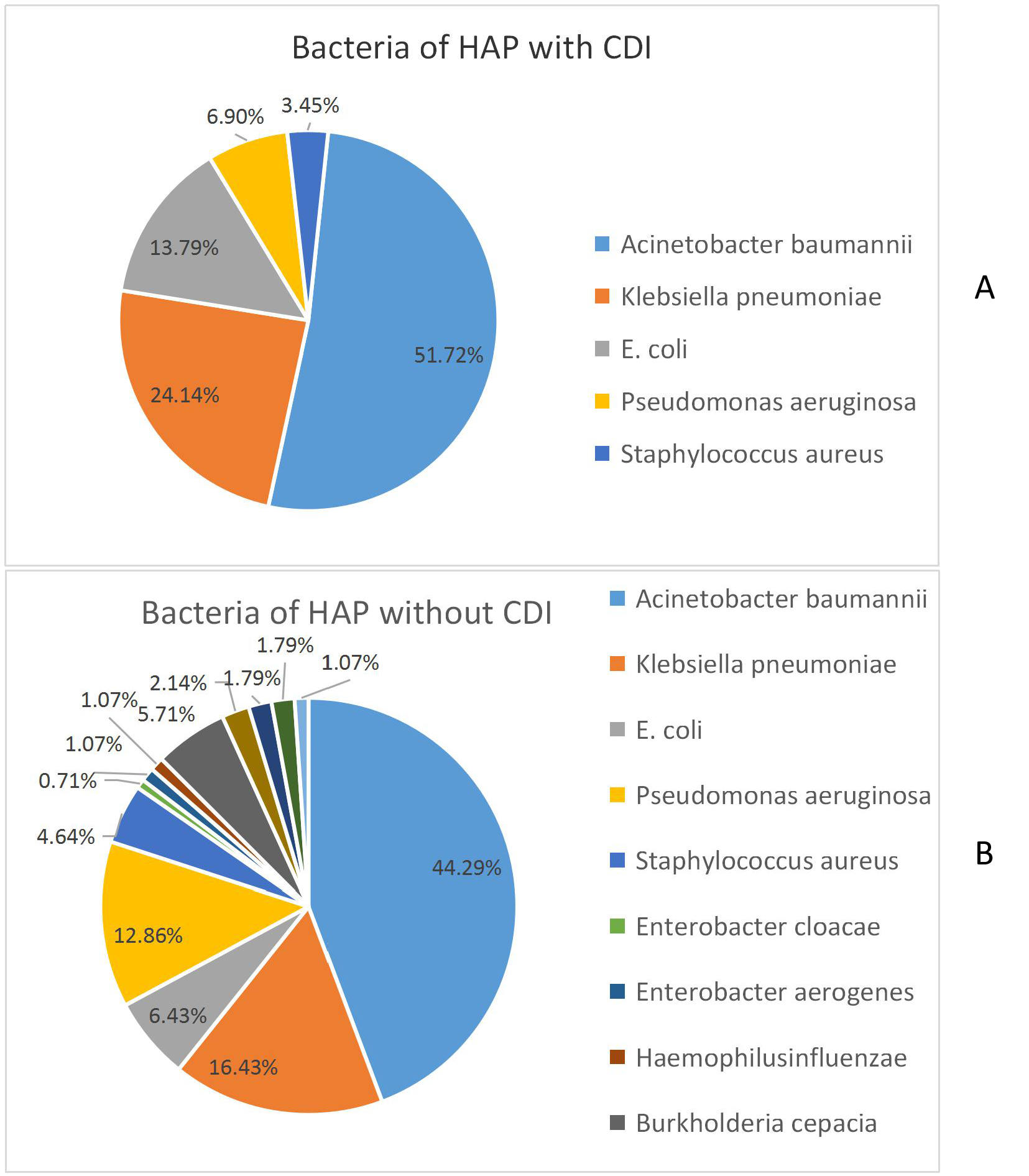

Supplement: Supplementary file 1 — Supplemental Fig. 1. The proportion of bacterial for HAP among HAP-CDI group (A) and HAP-nonCDI group (B) (TIFF 10795 kb) [file 15010_2017_1024_MOESM1_ESM.tif]

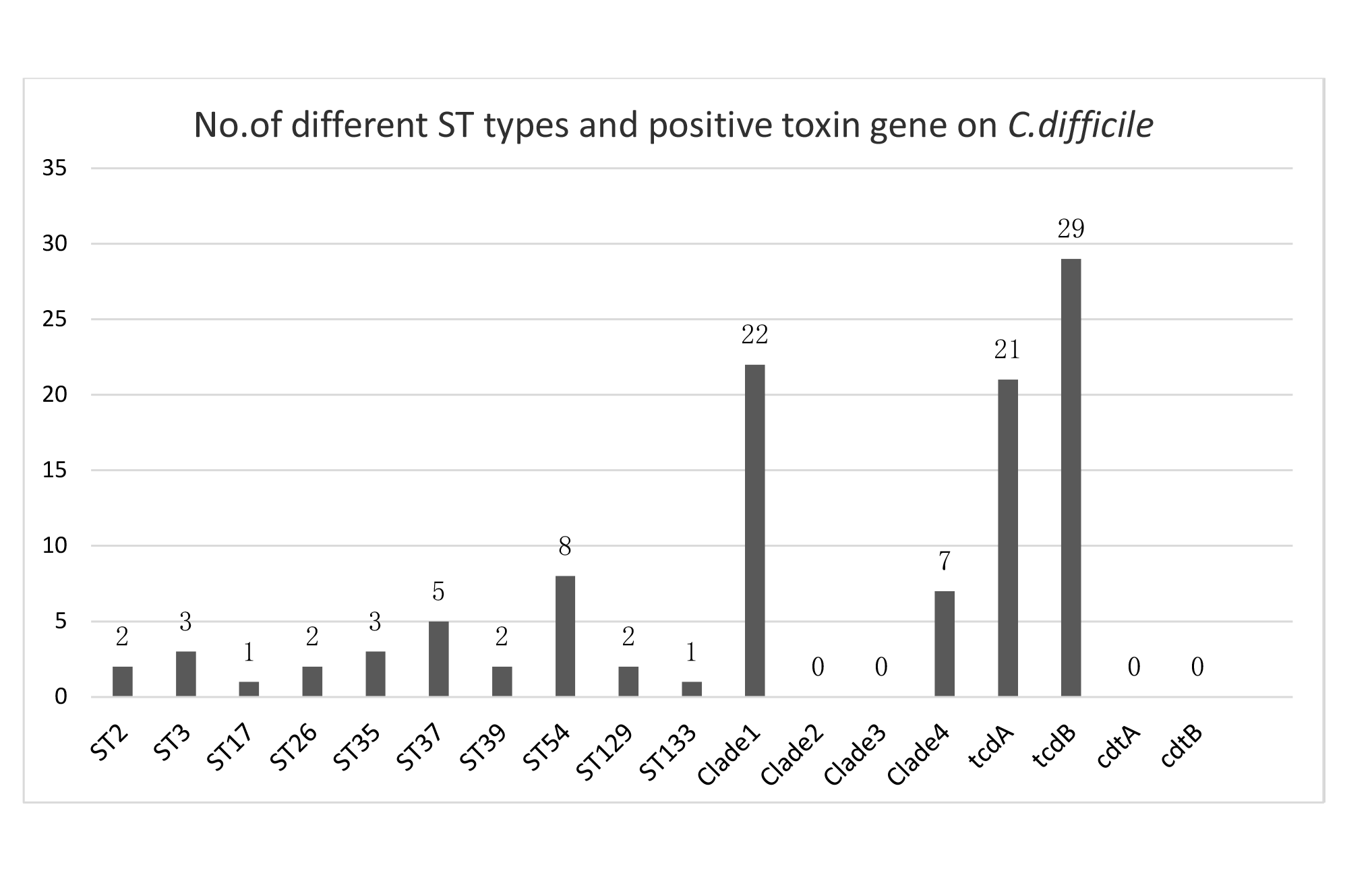

Supplement: Supplementary file 2 — Supplemental Fig. 2. Number of different MLST types and positive toxin gene on C. difficile (TIFF 8133 kb) [file 15010_2017_1024_MOESM2_ESM.tif]
