# Supplementary material for: Assessing the risk and disease burden of Clostridium difficile infection among patients with hospital-acquired pneumonia at a University Hospital in Central China
Source: Infection. 2017 May 11;45(5):621–8. doi: 10.1007/s15010-017-1024-1 (PMC5630651; doi:10.1007/s15010-017-1024-1)
Supplement: Supplementary file 3 — Supplementary material 3 (DOCX 13 kb) [file 15010_2017_1024_MOESM3_ESM.docx]

| **Antimicrobial category** | **Antimicrobial agents** | | | | |
| --- | --- | --- | --- | --- | --- |
| Aminoglycosides | Gentamicin | Amikacin |  |  |  |
| Antipseudomonal penicillins+inhibitors | Piperacillin-tazobactam | |  |  |  |
| Broad-spectrum cephalosporins+inhibitors | cefoperazone-sulbactam | |  |  |  |
| Carbapenems | Imipenem | Meropenem |  |  |  |
| Non-broad cephalosporins | Cefazolin | Cefuroxime |  |  |  |
| Broad -spectrum cephalosporins | Cefotaxime | ceftriaxone | Ceftazidime | Cefepime | Cefoselis |
| FIuoroqinoIones | Ciprofloxacin | Levofloxacin | Moxifloxacin |  |  |
| Glycopeptides | Vancomycin |  |  |  |  |
| Oxacephems | moxalactam |  |  |  |  |
| Nitroimidazoles | Tinidazole | Metronidazole |  |  |  |

Supplemental table 1: All the antimicrobial categories and agents of this study
